# Medical students’ perception of AI’s role in radiology before and after an AI-focused educational panel: a paired pre-post design

**DOI:** 10.1186/s12909-025-08319-9

**Published:** 2025-12-29

**Authors:** Nazir Sirajudeen, Nishika Bhatt, Ajay Patel, Gemma Fallouh, Anna Hyclová, Madhur Varadpande, Aryan Goel, Washsister Rajaratnam, Samiullah Dost

**Affiliations:** 1 University College London, London, United Kingdom; 2 King’s College London, London, United Kingdom; 3 Imperial College London, London, United Kingdom; 4 St George’s, University of London, London, United Kingdom; 5 Guy’s and St Thomas’ NHS Foundation Trust, London, United Kingdom

**Keywords:** Artificial intelligence, Medical student, Radiology, Education

## Abstract

**Background:**

Artificial intelligence (AI) is increasingly applied in clinical diagnostics, particularly in radiology, where it can assist with imaging triaging and anomaly detection. However, the integration of AI into medical education remains under researched. This study investigates the impact of an AI-focused panel discussion on medical students’ perceptions, knowledge, attitudes and concerns about AI in radiology.

**Methods:**

A paired pre-post design questionnaire comprising of 13 five-point Likert scale questions was administered to 40 medical students to complete before and after an AI-focused educational panel session at the International Radiology Undergraduate Symposium in London, United Kingdom on 24th November 2024. The questionnaire assessed four domains: 'Understanding of AI,' 'Attitudes Toward AI in Radiology,' 'AI Education in Medical School,' and 'Concerns About AI in the Future.' The primary outcome was to assess the change in students’ perceptions of AI’s role in radiology.

Differences between pre- and post-session responses were analysed using the Wilcoxon signed-rank test. The Hodges-Lehmann median difference, the effect size, r, and their corresponding 95% confidence intervals were calculated, and p-values were adjusted using the Holm-Bonferroni method.

**Results:**

Of the 81 eligible attendees, 40 (49.4%) completed the questionnaire (39 pre-session, 40 post-session). Students demonstrated significant improvements in their understanding of AI’s potential role in radiology (Z = 3.04, p = 0.002; Holm–Bonferroni = 0.029; median paired difference = 0.5, 95% CI 0.0–0.5; r = 0.49, 95% CI 0.25–0.68) and in their awareness of AI’s broader clinical applications (Z = 3.65, p < 0.001; Holm–Bonferroni = 0.0035; median paired difference = 0.5, 95% CI 0.5–1.0; r = 0.60, 95% CI 0.38–0.75). Participants expressed a more positive view of AI in healthcare overall, although concerns about AI replacing radiologists and insufficient AI education persisted.

**Conclusion:**

Educational interventions have the potential to improve medical students’ understanding and attitudes toward AI in radiology. Integrating structured AI education into undergraduate curricula may enhance AI literacy and better prepare future clinicians for an AI-enabled healthcare environment.

**Supplementary Information:**

The online version contains supplementary material available at 10.1186/s12909-025-08319-9.

## Background

Artificial intelligence (AI) is revolutionising healthcare at speed and is becoming integral to modern radiology, with AI-driven algorithms showing effectiveness in identifying abnormalities such as acute bleeds and fractures, often achieving diagnostic accuracy comparable to expert radiologists [1, 2]. These technologies can enable image triaging, report generation, streamlining radiology workflows and reducing diagnostic delays [3]. However, while AI promises to augment clinical efficiency and patient care, its integration into radiological practice also raises questions about workforce implications, ethical considerations, and the need for clinicians to adapt to rapidly evolving technology [4].

The integration of AI into medical education in the UK is still in its early stages, with limited representation in the formal curriculum across medical schools, leading to knowledge gaps regarding its practical implementation [5]. Most AI-related content is incorporated within broader digital health or elective extracurricular societies discussing foundational content rather than applied skills or domain-specific knowledge [6]. While there is growing recognition of AI’s relevance in healthcare, a standardised approach to AI education is lacking within the medical school curriculum, with students from across the globe reporting poor familiarisation of AI’s role in this field [7–9]. Recent consensus-based frameworks have begun outlining essential competencies for AI literacy within undergraduate medical education, highlighting the need for structured AI teaching [10].

Educational interventions, such as AI-focused educational panels, serve as a platform to bridge these knowledge gaps, providing students with evidence-based insights into AI’s functionalities and fostering a balanced understanding of its benefits and challenges. Prior research suggests that educational interventions significantly impact students’ acceptance of AI in medicine [11]. However, there remains a pressing need to assess whether targeted discussions can alleviate misconceptions, clarify the collaborative nature of AI and radiologists, and reduce concerns regarding job displacement. To our knowledge, no published studies have investigated medical students’ perceptions of AI in radiology before and after an educational intervention.

Therefore, we aim to explore the impact of an AI-focused educational panel discussion on medical students’ perceptions of AI in radiology. By assessing changes in students’ knowledge and attitudes before and after the intervention, we aim to identify a potential effective strategy for integrating AI education into medical curricula, ultimately enhancing students’ readiness to engage with AI technologies in their future clinical practice.

## Methods

### Study design

This study employed a paired pre-post design questionnaire distributed to participants attending an AI-focused educational panel session. Our study was conducted in accordance with the Strengthening the Reporting of Observational Studies in Epidemiology (STROBE) guidelines [12].

### Setting

The panel session took place at the International Radiology Undergraduate Symposium (IRUS), held at Guy’s Campus, King’s College London, London, United Kingdom (UK) on 24th November 2024. The panel featured a consultant radiologist, a radiology registrar, and a consultant cardiologist with a special interest in AI applications in cardiac radiology. All attendees of the IRUS conference, primarily medical students from London-based medical schools, were invited to participate.

The panel lasted approximately 60 minutes and consisted of an interactive question-and-answer session. The session was designed with three learning objectives: (1) to introduce students to current applications of AI in radiological practice, (2) to explore the collaborative relationship between clinicians and AI technologies, and (3) to address ethical and professional considerations surrounding AI integration into clinical workflows. The discussion was intended to encourage critical thinking about AI’s role in augmenting, rather than replacing, human expertise in radiology.

Participation in the study was voluntary and anonymous. The questionnaire was printed and distributed before the session, with clear instructions to complete only the pre-session section at that time. Participants were explicitly informed that only medical students from UK medical schools were eligible for inclusion in the study.

Following the conclusion of the panel session, participants were given time to complete the post-panel section of the questionnaire.

### Survey design

The questionnaire consisted of 13 five-point Likert scale questions, categorized into four domains: ‘Understanding of AI,’ ‘Attitudes Toward AI in Radiology,’ ‘AI Education in Medical School,’ and ‘Concerns About AI in the Future.’ Responses were scored as 1 (‘strongly disagree’), 2 (‘disagree’), 3 (‘neutral’), 4 (‘agree’) or 5 (‘strongly agree’).

The questionnaire was self-developed with reference to previously validated instruments identified in the literature and informed by key themes on artificial intelligence in medical education [8, 9]. These included participants’ understanding of AI use in radiology, their intentions to pursue radiology as a specialty, and their confidence in using AI. The questionnaire underwent internal review by a panel consisting of two doctors and eight medical students, followed by several iterative revisions to improve clarity, content validity, and alignment with the study objectives.

The questionnaire was printed on double-sided A4 paper, with one side designated for pre-panel responses and the other for post-panel responses. Additionally, the participants were asked to indicate their current year of study in medical school and their specialty of interest. A copy of the questionnaire is provided in Additional file 1.

### Statistical methods

Questionnaire data collected from the study were entered into a Microsoft Excel spreadsheet (Microsoft Corporation, Redmond, WA) for organisation and preliminary analysis. Simple descriptive statistics were calculated to summarise participant characteristics and questionnaire responses.

Ordinal data from 13 five-point Likert-scale questions were numerically coded from ‘1’ to ‘5’. This dataset was assessed for normality using the Shapiro–Wilk test and found to be non-parametric. To evaluate differences between pre- and post-panel questionnaire responses, the Wilcoxon signed-rank test was employed for each individual question item, as it is appropriate for paired, non-normally distributed data.

The Wilcoxon signed-rank test first calculates the absolute differences between paired pre- and post-panel values, ranks these differences by magnitude, and assigns a positive or negative sign to each rank based on the direction of change. A test statistic is then generated and compared against the expected distribution under the null hypothesis of no median difference. The Related Samples Hodges–Lehmann (HL) Median Difference was used to estimate the median paired difference and corresponding 95% confidence intervals (CI). Effect sizes, *r*, were reported with corresponding 95% CIs to quantify the magnitude of paired differences. To control for Type I error arising from multiple comparisons, p-values were adjusted using the Holm–Bonferroni method, maintaining the family-wise error rate at α = 0.05. A p-value of < 0.05 was considered statistically significant.

Descriptive statistics were generated using Microsoft Excel, and all analyses were performed using IBM Statistical Package for the Social Sciences (SPSS) 31.0 and R. Missing data were handled on a pairwise basis. Participants with missing pre- or post-session responses for a given Likert item were excluded from the Wilcoxon signed-rank analysis for that item only but were retained in analyses of other items they fully completed. All available data were included when calculating descriptive statistics.

### Outcomes

The primary outcome was to assess the change in participants’ perceptions and confidence regarding AI’s role in radiology following the educational panel session, evaluated through differences in paired Likert-scale responses between the pre- and post-session questionnaires.

Secondary outcomes assessed changes in specific domains of perception, including students’ understanding of AI applications in clinical practice, their attitudes toward the inclusion of AI education within the medical school curriculum, and their perspectives on pursuing a career in radiology given AI’s evolving role.

## Results

**Fig. 1:**
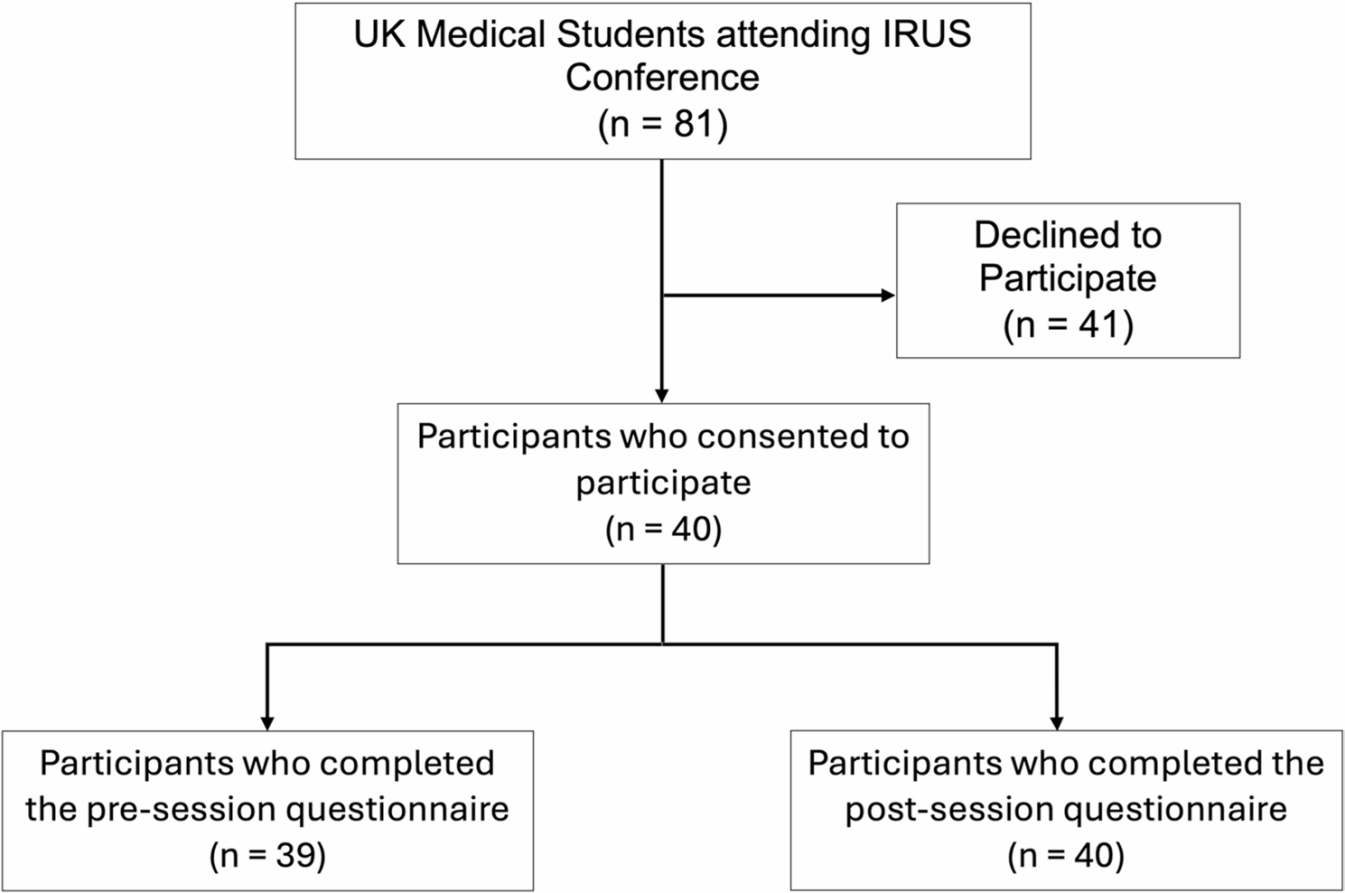
Participant flow diagram: Flow diagram illustrating participant recruitment and retention. Of 81 UK medical students attending the IRUS Conference, 40 consented to participate in the study. Thirty-nine participants completed the pre-session questionnaire, and 40 completed the post-session questionnaire

A total of 81 attendees were eligible and invited to participate in the study, of whom 40 completed the questionnaire, yielding a response rate of 49.4%. Of these, 39 participants completed the pre-session section and all 40 completed the post-session section. Fig. 1 below shows the participant flow diagram. The majority (65%) of the participants were from years 1–3 of medical school. Eighteen of the 40 participants (45%) had indicated radiology, either diagnostic or interventional, as a speciality of interest. Table 1 below shows the results for all participants.

**Table 1:** Wilcoxon signed-rank test results for pre- and post-session Likert scores. Bold indicates p < 0.05

Question	Domain Assessed	Pre-session mean	Post-session mean	Z-value	Median difference (Hodges-Lehmann, 95% CI)	Effect size (*r*)	*p*-value	Holm-Bonferroni corrected *p*-value
I understand the basic principles of AI	Understanding of AI	3.41	3.85	2.9235	0.50 [0.00 – 0.50]	0.47 [0.22 − 0.65]	**0.00346**	**0.03806**
I can understand where AI will be useful in my day-to-day medical practice	Understanding of AI	3.46	4.15	3.6454	0.50 [0.50 – 1.00]	0.60 [0.38 − 0.75]	**0.00027**	**0.00351**
AI will play a vital role in healthcare in general	Understanding of AI	3.92	4.30	2.3707	0.50 [0.00 – 0.50]	0.35 [0.06–0.60]	**0.01776**	0.17760
AI will replace many routine tasks currently performed by radiologists	Attitudes Toward AI in Radiology	3.18	3.08	−0.7136	0.00 [−0.50 – 0.50]	0.06 [0.01–0.39]	0.47549	1.00000
AI will make medical decision-making safer and more reliable	Attitudes Toward AI in Radiology	3.23	3.58	2.1656	0.50 [0.00 – 0.50]	0.36 [0.07–0.61]	**0.03034**	0.27153
I understand AI’s potential role in radiology	Attitudes Toward AI in Radiology	3.77	4.20	3.0378	0.50 [0.00 – 0.50]	0.49 [0.25–0.68]	**0.00238**	**0.02856**
I expect AI will reduce patient interaction in radiology	Attitudes Toward AI in Radiology	2.71	2.51	−1.6036	−0.50 [−0.50 − 0.00]	0.25 [0.02–0.55]	0.10881	0.65286
AI is adequately taught in the medical school curriculum	AI Education in Medical School	1.51	1.53	0.2774	0.00 [0.00 – 0.00]	0.01 [0.00–0.34]	0.78151	1.00000
Learning about AI is essential to staying relevant in future medical practice	AI Education in Medical School	4.26	3.93	−2.1679	0.00 [−0.50 – 0.00]	0.28 [0.03–0.54]	**0.03017**	0.27153
AI will replace many specialties in my lifetime	Concerns About AI in the Future	2.10	2.15	−0.0539	0.00 [−0.50 – 0.50]	0.02 [0.00–0.37]	0.95704	1.00000
AI is most likely to affect the role of radiologists out of all other clinical specialties	Concerns About AI in the Future	3.13	3.05	−0.2243	0.00 [−0.50 – 0.50]	0.09 [0.01–0.42]	0.82252	1.00000
AI will make me less likely to pursue a career in radiology	Concerns About AI in the Future	2.00	1.95	−0.4407	0.00 [−0.50 – 0.00]	0.10 [0.01–0.41]	0.65942	1.00000
The role of radiologists will broadly remain unchanged despite AI advancements	Concerns About AI in the Future	2.49	2.93	1.9874	0.50 [0.00 – 1.00]	0.31 [0.03–0.57]	**0.04688**	0.32816

**Fig. 2:**
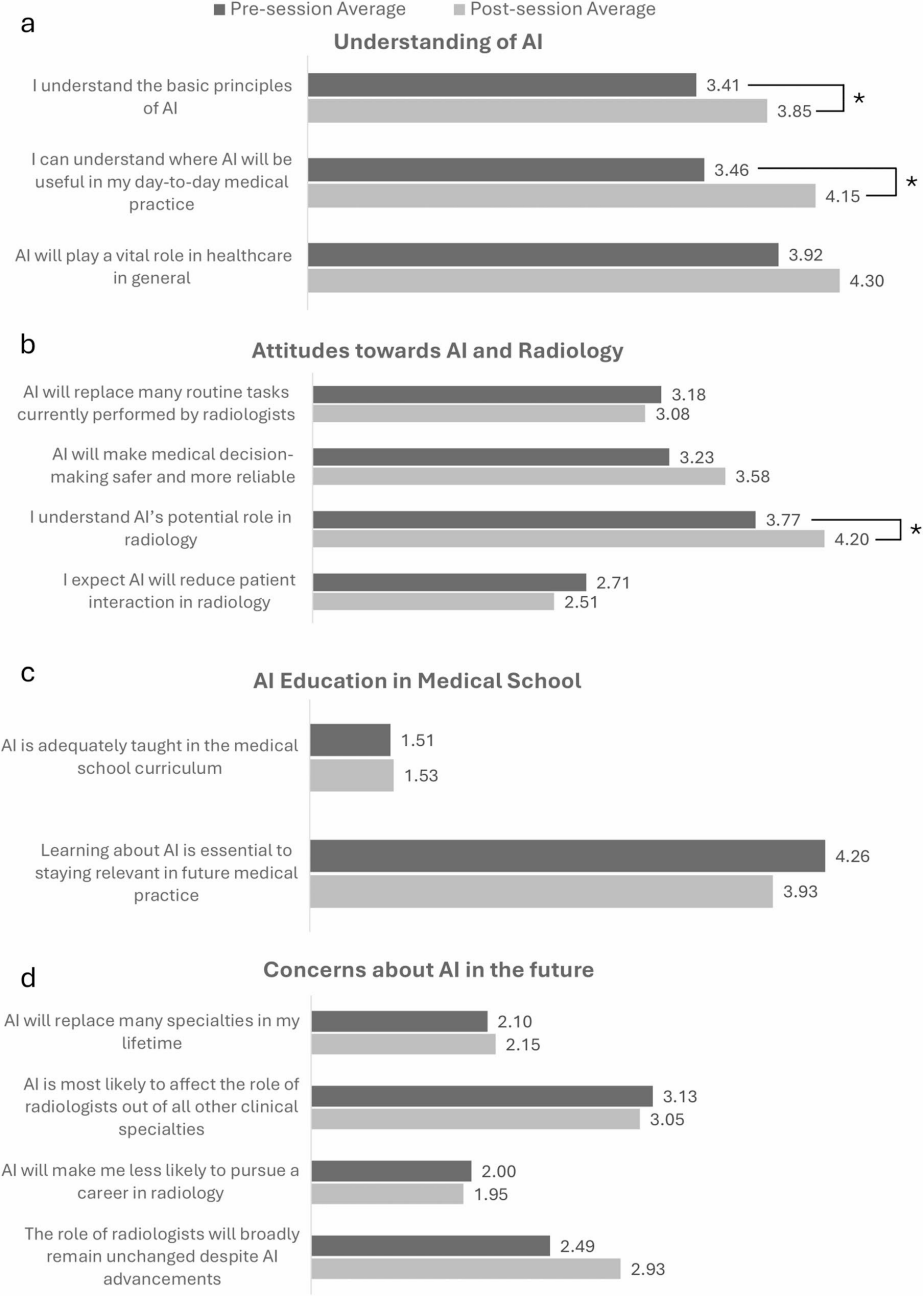
Mean average Likert scores for each question pre- and post-session across four domains: (a) Understanding of AI, (b) Attitudes towards AI and Radiology, (c) AI Education in Medical School, and (d) Concerns about AI in the Future. Bar charts display the mean Likert score for each item before and after the teaching session. An asterisk (*) indicates a statistically significant difference between pre- and post-session scores after applying the Holm-Bonferroni correction (p < 0.05).

Before the panel session, the statement which yielded the highest agreement with the students was ‘Learning about AI is essential to staying relevant in future medical practice’ (4.26) and the statement which yielded the strongest disagreement was ‘AI is adequately taught in the medical school curriculum’ (1.51). After the session, the statement which yielded the highest agreement was ‘AI will play a vital role in healthcare in general’ (4.30) and the statement which yielded the strongest disagreement was ‘AI is adequately taught in the medical school curriculum’ (1.53). Fig. 2 below shows the mean scores for each question before and after the panel session.

**Fig. 3:**
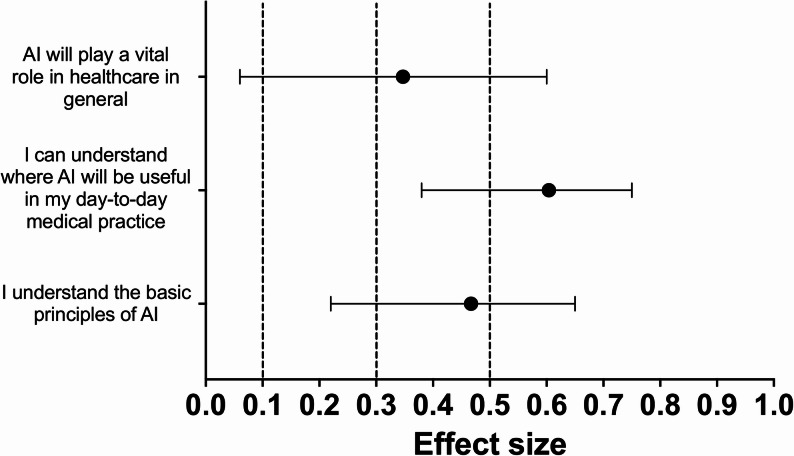
**Effect sizes for questions in the ‘Understanding of AI’ domain:** Forest plot illustrating the effect sizes (*r*) derived from Wilcoxon signed-rank tests for each question within the ‘Understanding of AI’ domain

**Fig. 4:**
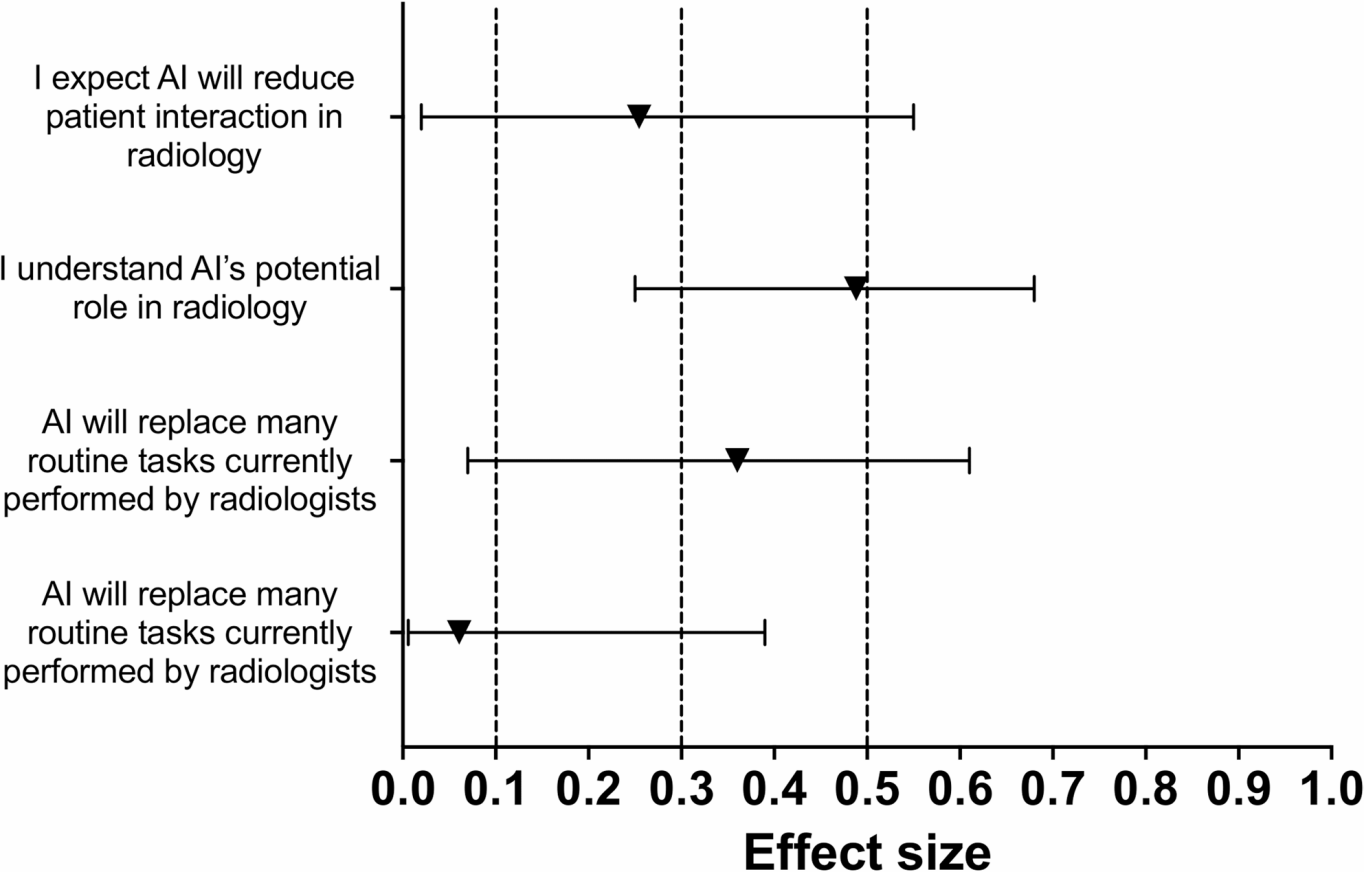
**Effect sizes for questions in the ‘Attitudes Toward AI in Radiology’ domain:** Forest plot illustrating the effect sizes (*r*) derived from Wilcoxon signed-rank tests for each question within the ‘Attitudes Toward AI in Radiology’ domain

**Fig. 5:**
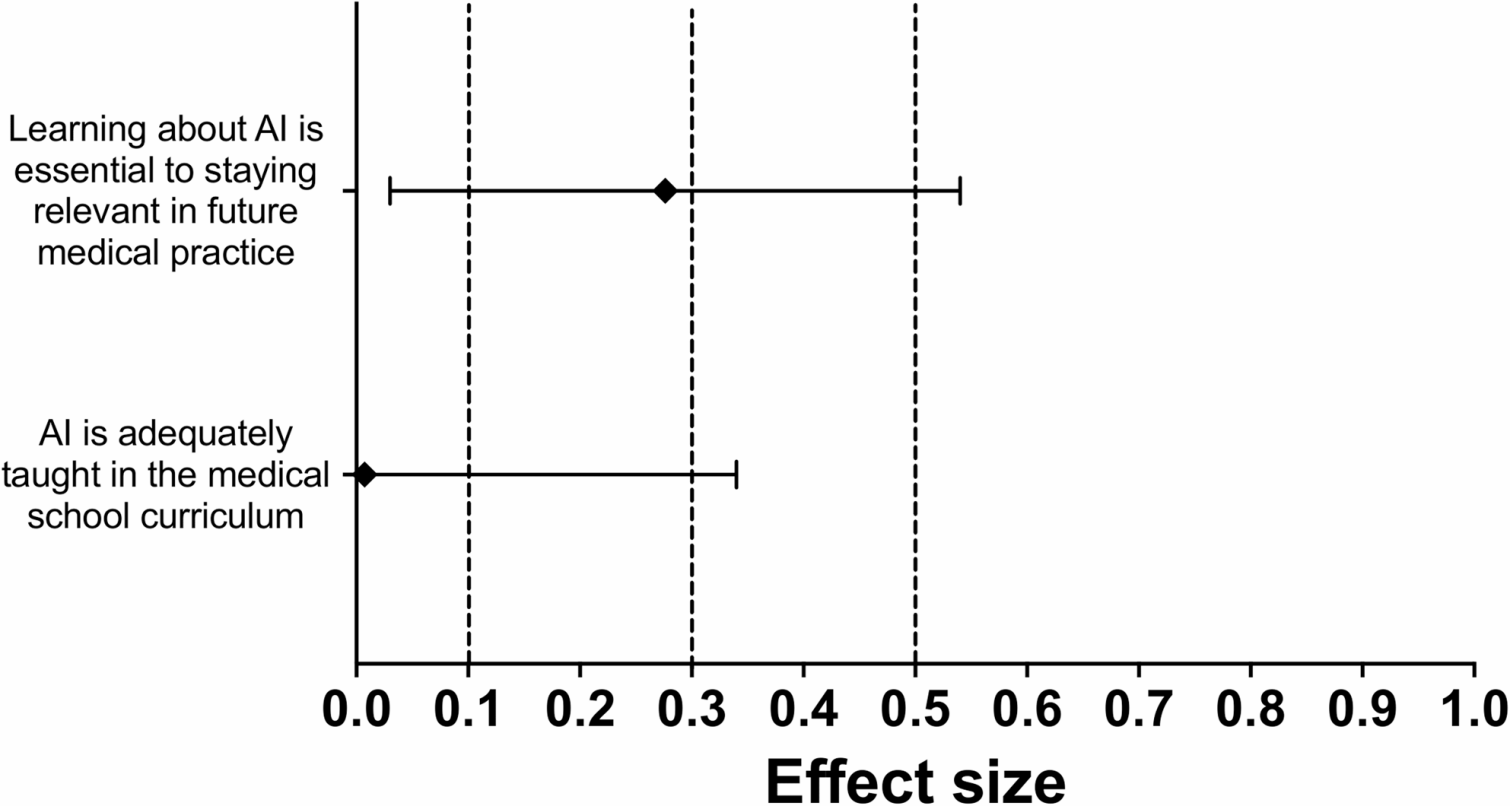
**Effect sizes for questions in the ‘AI Education in Medical School’ domain: **Forest plot illustrating the effect sizes (*r*) derived from Wilcoxon signed-rank tests for each question within the 'AI Education in Medical School’ domain

**Fig. 6:**
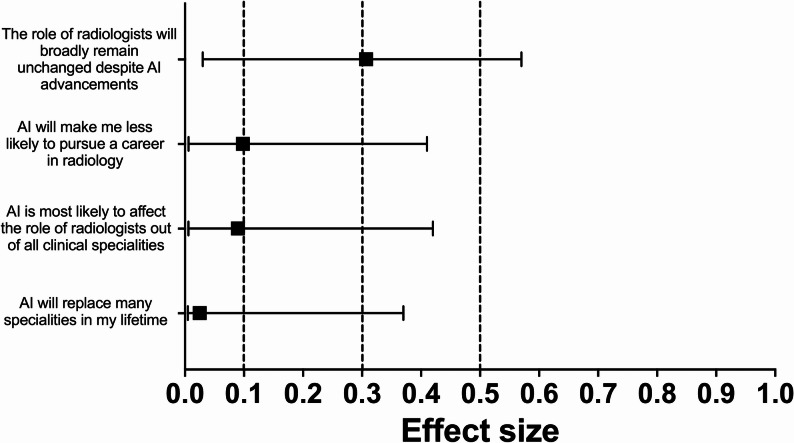
**Effect sizes for questions in the 'Concerns About AI in the Future’ domain:** Forest plot illustrating the effect sizes (*r*) derived from Wilcoxon signed-rank tests for each question within the 'Concerns About AI in the Future’ domain

Seven of the 13 statements (54%) showed a statistically significant difference in agreement following the educational session. After applying the Holm–Bonferroni correction, a statistically significant increase in agreement remained for three statements (23%). The greatest difference was observed for the statement “I can understand where AI will be useful in my day-to-day medical practice” (Z = 3.65, *p* < 0.001; Holm–Bonferroni = 0.0035; HL median difference = 0.50, 95% CI 0.50–1.00; *r* = 0.60, 95% CI 0.38–0.75). The other two statements that remained significant after correction were “I understand AI’s potential role in radiology” (Z = 3.04, *p* = 0.002; Holm–Bonferroni = 0.0286; HL median difference = 0.50, 95% CI 0.00–0.50; *r* = 0.49, 95% CI 0.25–0.68) and “I understand the basic principles of AI” (Z = 2.92, *p* = 0.003; Holm–Bonferroni = 0.0381; HL median difference = 0.50, 95% CI 0.00–0.50; *r* = 0.47, 95% CI 0.22–0.65). These findings reflect measurable changes in students’ perceptions and awareness of AI’s role and applications in radiology. No statement showed a statistically significant decrease in agreement after correction. Fig. 3, 4, 5 and 6 below show the effect sizes, *r*, for items within each of the four domains.

## Discussion

The integration of AI into radiology represents a transformative shift in medical practice, necessitating a corresponding evolution in medical education. Despite AI’s growing role in diagnostic imaging, many medical students lack sufficient exposure to AI-related concepts, leading to uncertainty, misconceptions, and apprehension regarding its impact on their future careers. Although studies indicate that medical students recognise AI’s growing importance in clinical practice, concerns persist regarding its impact on employment prospects, ethical challenges, and the risk of over-reliance on technology. In the absence of standardised AI education, students’ understanding is frequently influenced by media narratives rather than formal instruction, contributing to misconceptions about AI’s role in radiology [13]. This underscores the need for structured educational frameworks that offer a balanced perspective on AI’s clinical applications, limitations, and ethical implications [14].

Sit et al. conducted a multi-institutional study across 19 UK medical schools to examine the influence of AI on medical students’ intentions to pursue a career in radiology [8]. Approximately half of the students were less likely to pursue this field due to the perceived success of AI implementation in healthcare. Galán and Portero found that while 81.2% of Spanish medical students acknowledged AI’s transformative role in radiology, 55.6% of those considering radiology as their preferred speciality were concerned about AI’s potential impact on their future roles [15]. Such misconceptions could further exacerbate the UK’s ongoing radiology workforce shortages, deterring students from pursuing the specialty [8, 16].

Although some students had apprehensive views of AI’s implementation into radiology, Hashmi et al. reported encouraging findings in a survey of 149 radiology trainees [17]. A majority of trainees expressed enthusiasm for involvement in AI-based projects (79.9%) and demonstrated a general interest in understanding its impact on radiology (83.7%). However, only one respondent reported having received formal AI education, while 98.7% indicated that structured AI training should be incorporated into the curriculum [17].

Ensuring that students receive structured AI teaching could enhance their preparedness to work alongside AI-driven technologies while mitigating misconceptions. Medical students are more inclined than qualified clinicians to perceive AI as a threat, potentially due to limited familiarity with its current practical applications in radiology [18]. Conversely, radiologists view AI’s function to augment rather than replace, with less than 20% of radiologists amongst a cohort in Germany trusting the results of AI alone [19–21]. Existing literature indicates that UK medical students possess a comparatively lower level of knowledge about AI than their international counterparts [22]. However, students within the UK who had received AI training were less likely to rule out a career within radiology [8].

In our study, participants showed an increase in agreement that the role of radiologists is expected to remain largely unchanged despite advances in AI. Additionally, there was a small, but statistically non-significant, decrease in apprehension toward pursuing a career in radiology due to AI following the panel session. These findings suggest an increased understanding and appreciation of AI as a complementary tool to enhance radiological practice. This emphasises the importance of integrating comprehensive AI education into the undergraduate curricula to promote AI literacy and alleviate concerns regarding the perceived threat of AI replacing radiologists. Radiologists themselves, given their expertise within the field, can provide valuable input and advocate for the integration of AI education within their institutions.

This study explores the impact of a single targeted educational panel on medical students’ perceptions of AI in radiology, offering insights into how structured educational interventions can foster AI literacy among future healthcare professionals. There was a statistically significant improvement in understanding of AI and its integration into clinical practice following the interactive panel intervention, indicating its potential value within the medical school curriculum. However, the magnitude of change was relatively modest. These findings suggest that educational panel sessions may be a useful component of medical training; however, further research is needed to evaluate the optimal intervention format and timeframe. Insights from this single educational panel discussion might serve as a foundation for future work exploring more longitudinal approaches to AI in radiology education, rather than one-off sessions.

There was no statistically significant change in opinions on AI replacing radiologists and other specialties, its impact on patient interaction, and the adequacy of AI in undergraduate medical education. These findings suggest an ongoing ambiguity among students regarding the role of AI in shaping a career in radiology. Notably, statistically significant changes in opinion were more often associated with subjective statements based on students’ personal understanding, whereas statements that did not show significant changes were generally more objective reflections on AI’s clinical applications, potentially due to students’ limited knowledge of the field. This may further point towards a need for implementation of longitudinal educational interventions into the medical school curriculum rather than a one-off panel, to account for the students’ limited prior exposure to AI in radiology.

Beyond radiology, this study has broader implications for AI literacy across multiple medical specialties. While radiology is at the forefront of AI adoption, AI-driven technologies are becoming increasingly prevalent in fields such as pathology, dermatology, and oncology [2, 23–25]. Ensuring that medical students across disciplines are equipped with the knowledge and critical thinking skills necessary to engage with AI is essential for the safe and effective integration of these technologies into clinical practice. This study offers insights into how structured educational strategies can be applied across various specialties, fostering a generation of AI-literate physicians who can leverage AI to enhance patient care while maintaining a strong foundation in clinical decision-making.

Overall, by demonstrating how expert-led discussions influence students’ perceptions, this study highlights the need to integrate AI-focused educational interventions into medical training. The findings provide a framework for incorporating AI-related content into radiology education through expert-led panel discussions. The significant yet modest impact of an expert-led panel discussion on students’ perceptions suggests that delivering such interventions serially over an extended period may play a valuable role in aligning medical education with the evolving landscape of AI and its implications for radiology. Future expert-led teaching incorporated into the medical curriculum would be beneficial in ensuring that all future clinicians develop the necessary competencies to engage effectively with AI-assisted diagnostic tools. This could also influence students’ perceptions of AI replacing radiologists and help promote an understanding of AI as a tool to enhance diagnostics and workflow optimisation rather than as a replacement for radiologists.

### Limitations

There are several limitations to this study. First, the potential for selection bias must be acknowledged. The symposium was explicitly advertised with an explicit focus on AI in radiology, and attendance by medical students was voluntary. Consequently, it is likely that participants with a pre-existing interest in the subject were disproportionately represented, potentially limiting the generalisability of the findings. Moreover, the relatively small sample size and the London-based setting may not translate to the opinions of medical students from other regions. Future work should explore educational interventions involving a larger and more diverse cohort, including both national and international medical students, to enhance the applicability and relevance of the results.

The questionnaire was not formally validated or piloted due to logistical and time constraints. However, its contents were informed by existing literature to ensure relevance of the data collected. As a result, certain areas of interest may not have been addressed, and some questions may require further clarification. Future iterations of the questionnaire may help refine the items posed and enhance the questionnaire’s comprehensiveness.

In addition, as all participants attended the panel session, there was no control or comparison group. This limits the ability to determine whether the observed changes in perceptions were directly attributable to the session itself, or if they are influenced by other external factors. Future work should focus on incorporating a control group to clarify the influence of these educational sessions.

Furthermore, the panel is not fully representative of all professional groups involved in the implementation of AI in radiology, notably computer scientists and radiographers. Although experts in cardiovascular imaging were represented, future sessions would benefit from broader inclusion across a breadth of radiological subspecialties to ensure a more inclusive and multidisciplinary perspective. This would enable a more comprehensive understanding of the diverse challenges and considerations associated with AI implementation across radiology.

Finally, the panel intervention was structured such that students directed the discussion by posing their own questions. This approach may have influenced the number of statistically significant changes observed in survey responses. In contrast, an expert-led intervention, such as a lecture series, could offer targeted teaching on AI’s role in radiology, potentially improving understanding and yielding more pronounced changes. Although several statements demonstrated statistically significant changes, the magnitude of change in Likert scores was modest, indicating that shifts in perception were relatively small. Moreover, as post-session surveys were administered immediately after the panel, the observed gains could reflect short-term changes in perception rather than sustained improvements. This may reflect the limitations of a single panel discussion in achieving substantial changes in viewpoint and further supports the case for alternative formats involving multiple interventions delivered over an extended period.

## Conclusion

Ultimately, this study underscores the importance of structured AI education in preparing future healthcare professionals for an AI-augmented medical landscape. By evaluating the impact of a targeted AI-focused educational panel, it provides evidence on how educational interventions can shape students’ readiness to engage with AI in clinical settings. The findings serve as a foundation for developing standardised AI education strategies, ensuring that medical graduates are equipped to navigate the evolving intersection of AI and clinical practice.

## Supplementary Information

Supplementary Material 1. AI panel questionnaire: This document contains the full questionnaire used during the AI panel session, including all items presented to participants before and after the session, using a 5-point Likert scale.

## Data Availability

The datasets used and/or analysed during the current study are available from the corresponding author on reasonable request.

## Abbreviations

AI: Artificial Intelligence
STROBE: Strengthening the Reporting of Observational Studies in Epidemiology
IRUS: International Radiology Undergraduate Symposium
UK: United Kingdom
HL: Hodges-Lehmann
CI: Confidence Interval
SPSS: Statistical Package for the Social Sciences

## References

[1] Topol EJ. Deep medicine: how artificial intelligence can make healthcare human again. First edition. New York: Basic Books; 2019.

[2] Pesapane F, Codari M, Sardanelli F. Artificial intelligence in medical imaging: threat or opportunity? Radiologists again at the forefront of innovation in medicine. Eur Radiol Exp. 2018;2(1):35. 30353365 10.1186/s41747-018-0061-6 PMC6199205

[3] Adenova G, Kausova G, Saliev T, Zhukov Y, Ospanova D, Dushimova Z, et al. Optimization of radiology diagnostic services for patients with stroke in multidisciplinary hospitals. Mater Sociomed. 2024;36(2):160–72. 39712327 10.5455/msm.2024.36.160-172 PMC11663002

[4] Langlotz CP. The future of AI and informatics in radiology: 10 predictions. Radiology. 2023;309(1):e231114. 37874234 10.1148/radiol.231114 PMC10623186

[5] Park CJ, Yi PH, Siegel EL. Medical student perspectives on the impact of artificial intelligence on the practice of medicine. Curr Probl Diagn Radiol. 2021;50(5):614–9. 32680632 10.1067/j.cpradiol.2020.06.011

[6] Paranjape K, Schinkel M, Nannan Panday R, Car J, Nanayakkara P. Introducing artificial intelligence training in medical education. JMIR Med Educ. 2019;5(2):e16048. 31793895 10.2196/16048 PMC6918207

[7] McLennan S, Meyer A, Schreyer K, Buyx A. German medical students´ views regarding artificial intelligence in medicine: a cross-sectional survey. PLoS Digit Health. 2022;1(10):e0000114. 36812635 10.1371/journal.pdig.0000114 PMC9931368

[8] Sit C, Srinivasan R, Amlani A, Muthuswamy K, Azam A, Monzon L, et al. Attitudes and perceptions of UK medical students towards artificial intelligence and radiology: a multicentre survey. Insights Imaging. 2020;11(1):14. 32025951 10.1186/s13244-019-0830-7 PMC7002761

[9] Allam RM, Abdelfatah D, Khalil MIM, Elsaieed MM, El Desouky ED. Medical students and house officers’ perception, attitude and potential barriers towards artificial intelligence in Egypt, cross sectional survey. BMC Med Educ. 2024;24(1):1244. 39482613 10.1186/s12909-024-06201-8 PMC11529482

[10] Car J, Ong QC, Erlikh Fox T, Leightley D, Kemp SJ, Švab I, et al. The digital health competencies in medical education framework: an international consensus statement based on a Delphi study. JAMA Netw Open. 2025;8(1):e2453131. 39888625 10.1001/jamanetworkopen.2024.53131

[11] Jackson P, Ponath Sukumaran G, Babu C, Tony MC, Jack DS, Reshma VR, et al. Artificial intelligence in medical education - perception among medical students. BMC Med Educ. 2024;24(1):804. 39068482 10.1186/s12909-024-05760-0 PMC11283685

[12] von Elm E, Altman DG, Egger M, Pocock SJ, Gøtzsche PC, Vandenbroucke JP, et al. The strengthening the reporting of observational studies in epidemiology (STROBE) statement: guidelines for reporting observational studies. PLoS Med. 2007;4(10):e296. 17941714 10.1371/journal.pmed.0040296 PMC2020495

[13] Tejani AS, Elhalawani H, Moy L, Kohli M, Kahn CE. Artificial intelligence and radiology education. Radiol Artif Intell. 2022;5(1):e220084. 36721409 10.1148/ryai.220084 PMC9885376

[14] Doumat G, Daher D, Ghanem NN, Khater B. Knowledge and attitudes of medical students in Lebanon toward artificial intelligence: a National survey study. Front Artif Intell. 2022;5:1015418. 36406470 10.3389/frai.2022.1015418 PMC9668059

[15] Caparrós Galán G, Sendra Portero F. Medical students’ perceptions of the impact of artificial intelligence in radiology. Radiologia. 2021;S0033-8338(21):00084 – 9. 10.1016/j.rxeng.2021.03.008 36402537

[16] Clinical radiology census reports |. The Royal College of Radiologists. Available from: https://www.rcr.ac.uk/news-policy/policy-reports-initiatives/clinical-radiology-census-reports/. [cited 2025 May 23]

[17] Hashmi OU, Chan N, de Vries CF, Gangi A, Jehanli L, Lip G. Artificial intelligence in radiology: trainees want more. Clin Radiol. 2023;78(4):e336-41. 36746724 10.1016/j.crad.2022.12.017

[18] Van Hoek J, Huber A, Leichtle A, Härmä K, Hilt D, Von Tengg-Kobligk H, et al. A survey on the future of radiology among radiologists, medical students and surgeons: students and surgeons tend to be more skeptical about artificial intelligence and radiologists may fear that other disciplines take over. Eur J Radiol. 2019;121:108742. 31734640 10.1016/j.ejrad.2019.108742

[19] Jha S, Topol EJ. Adapting to artificial intelligence: radiologists and pathologists as information specialists. JAMA. 2016;316(22):2353–4. 27898975 10.1001/jama.2016.17438

[20] Santomartino SM, Yi PH. Systematic review of radiologist and medical student attitudes on the role and impact of AI in radiology. Acad Radiol. 2022;29(11):1748–56. 35105524 10.1016/j.acra.2021.12.032

[21] Jungmann F, Jorg T, Hahn F, Santos DP, dos, Jungmann SM, Düber C, et al. Attitudes toward artificial intelligence among Radiologists, IT Specialists, and industry. Acad Radiol. 2021;28(6):834–40. 32414637 10.1016/j.acra.2020.04.011

[22] Amiri H, Peiravi S, rezazadeh shojaee S, sara, Rouhparvarzamin M, Nateghi MN, Etemadi MH, et al. Medical, dental, and nursing students’ attitudes and knowledge towards artificial intelligence: a systematic review and meta-analysis. BMC Med Educ. 2024;24(1):412. 38622577 10.1186/s12909-024-05406-1 PMC11017500

[23] Hogarty DT, Su JC, Phan K, Attia M, Hossny M, Nahavandi S, et al. Artificial intelligence in dermatology-where we are and the way to the future: a review. Am J Clin Dermatol. 2020;21(1):41–7. 31278649 10.1007/s40257-019-00462-6

[24] Shafi S, Parwani AV. Artificial intelligence in diagnostic pathology. Diagn Pathol. 2023;18(1):109. 37784122 10.1186/s13000-023-01375-z PMC10546747

[25] Shimizu H, Nakayama KI. Artificial intelligence in oncology. Cancer Sci. 2020;111(5):1452–60. 32133724 10.1111/cas.14377 PMC7226189

