# Supplementary material for: Medical students’ perception of AI’s role in radiology before and after an AI-focused educational panel: a paired pre-post design
Source: BMC Med Educ. 2025 Dec 29;25:1735. doi: 10.1186/s12909-025-08319-9 (PMC12751800; doi:10.1186/s12909-025-08319-9)
Supplement: Supplementary file 1 — Supplementary Material 1. AI panel questionnaire: This document contains the full questionnaire used during the AI panel session, including all items presented to participants before and after the session, using a 5-point Likert scale. [file 12909_2025_8319_MOESM1_ESM.docx]

We are conducting research on how an AI-focused educational panel may shift medical students’ perception of the impact of AI on radiology. We kindly ask participants to complete the questionnaire before and after the panel. Participation in this study is **voluntary** and all data will be **anonymised** at the point of collection.

Pre-session questionnaire

Speciality of Interest:

Medical school year (e.g. Year 4):

|  | Strongly Disagree | Disagree | Neutral | Agree | Strongly Agree |
| --- | --- | --- | --- | --- | --- |
| I understand the basic principles of AI |  |  |  |  |  |
| I can understand where AI will be useful in my day-to-day medical practice |  |  |  |  |  |
| AI will play a vital role in healthcare in general |  |  |  |  |  |
| AI will replace many routine tasks currently performed by radiologists |  |  |  |  |  |
| AI will make medical decision-making safer and more reliable |  |  |  |  |  |
| I understand AI’s potential role in radiology |  |  |  |  |  |
| I expect AI will reduce patient interaction in radiology |  |  |  |  |  |
| AI is adequately taught in the medical school curriculum |  |  |  |  |  |
| Learning about AI is essential to staying relevant in future medical practice |  |  |  |  |  |
| AI will replace many specialties in my lifetime |  |  |  |  |  |
| AI is most likely to affect the role of radiologists out of all other clinical specialities |  |  |  |  |  |
| AI will make me less likely to pursue a career in radiology |  |  |  |  |  |
| The role of radiologists will broadly remain unchanged despite AI advancements |  |  |  |  |  |

Any other comments:

Post-session questionnaire

|  | Strongly Disagree | Disagree | Neutral | Agree | Strongly Agree |
| --- | --- | --- | --- | --- | --- |
| I understand the basic principles of AI |  |  |  |  |  |
| I can understand where AI will be useful in my day-to-day medical practice |  |  |  |  |  |
| AI will play a vital role in healthcare in general |  |  |  |  |  |
| AI will replace many routine tasks currently performed by radiologists |  |  |  |  |  |
| AI will make medical decision-making safer and more reliable |  |  |  |  |  |
| I understand AI’s potential role in radiology |  |  |  |  |  |
| I expect AI will reduce patient interaction in radiology |  |  |  |  |  |
| AI is adequately taught in the medical school curriculum |  |  |  |  |  |
| Learning about AI is essential to staying relevant in future medical practice |  |  |  |  |  |
| AI will replace many specialties in my lifetime |  |  |  |  |  |
| AI is most likely to affect the role of radiologists out of all other clinical specialities |  |  |  |  |  |
| AI will make me less likely to pursue a career in radiology |  |  |  |  |  |
| The role of radiologists will broadly remain unchanged despite AI advancements |  |  |  |  |  |

Any other comments:
